# The Association Between Metabolic Syndrome and Frailty in Healthy Community-Dwelling Older Adults

**DOI:** 10.1093/geroni/igab046.2049

**Published:** 2021-12-17

**Authors:** A R M Saifuddin Ekram, Sara Espinoza, Michael Ernst, Lawrence Beilin, Nigel P Stocks, Joanne Ryan, Robyn Woods

**Affiliations:** 1 Monash University, Monash University, Melbourne, Victoria, Australia; 2 University of Texas Health Science Center San Antonio, San Antonio, Texas, United States; 3 University of Iowa, Iowa City, Iowa, United States; 4 The University of Western Australia (M570), Perth, Western Australia, Australia; 5 University of Adelaide, Adelaide, South Australia, Australia; 6 Monash University, Melbourne, Victoria, Australia

## Abstract

This study examined the association between metabolic syndrome (MetS) and frailty status in relatively healthy community-dwelling older adults. Participants included 19,114 individuals from the “ASPirin in Reducing Events in the Elderly” (ASPREE) trial. The diagnostic criteria for MetS were according to the International Diabetes Federation Task Force on Epidemiology and Prevention and the American Heart Association/National Heart, Lung, and Blood Institute (2009); and comprised any three of five parameters: waist circumference, triglycerides, fasting blood glucose, high-density lipoprotein cholesterol or hypertension. Frailty and prefrailty were defined using a modified Fried phenotype (FP) comprising exhaustion, body mass index, grip strength, gait speed and physical activity and a deficit accumulation frailty index (FI) of 66 items. The association between MetS and frailty was examined using multinomial logistic regression. At baseline, 51.1% of participants met the criteria of MetS; of those, 41.8% and 2.5 % were prefrail and frail, respectively, according to Fried phenotype, while 49.6% and 11.8 % were prefrail and frail, respectively, according to FI. MetS at baseline was associated with an increased likelihood of prefrailty (RRR: 1.25; 95% CI: 1.17, 1.33) and frailty (RRR: 1.60; 95% CI: 1.28, 2.01) compared to no frailty after adjustment for potential confounders according to Fried phenotype, while the association was stronger for prefrailty (RRR: 2.74; 95% CI: 2.55, 2.94) and frailty (RRR: 5.30; 95% CI: 4.60, 6.11) according to FI. Overall, at baseline, more than half of the participants had MetS, and the presence of MetS was significantly associated with pre-frailty and frailty.

